# Hyperglycemia in a NOD Mice Model of Type-I Diabetes Aggravates Collagenase-Induced Intracerebral Hemorrhagic Injury

**DOI:** 10.3390/biomedicines12081867

**Published:** 2024-08-15

**Authors:** Qasim M. Alhadidi, Kevin M. Nash, Ghaith A. Bahader, Emily Zender, Marcia F. McInerney, Zahoor A. Shah

**Affiliations:** 1 Department of Medicinal and Biological Chemistry, College of Pharmacy and Pharmaceutical Sciences, University of Toledo, Toledo, OH 43606, USA; 2 Department of Pharmacy, Al-Yarmok University College, Diyala 21163, Iraq; 3 Department of Pharmacology, College of Pharmacy and Pharmaceutical Sciences, University of Toledo, Toledo, OH 43606, USA

**Keywords:** diabetes, hemorrhage, NOD, neuroinflammation, oxidative stress

## Abstract

Background: Intracerebral hemorrhage (ICH) is a severe type of stroke with high mortality. Persistent hyperglycemia following ICH is linked to deteriorated neurological functions and death. However, the exacerbating effect of hyperglycemia on ICH injury at the molecular level is still unclear. Therefore, this study explores the impact of diabetes on ICH injury using a non-obese diabetic (NOD) mouse model of type I diabetes mellitus. Methods: NOD and non-diabetic (non-obese resistant) mice subjected to ICH by intrastriatal injection of collagenase were sacrificed three days following the ICH. Brains were collected for hematoma volume measurement and immunohistochemistry. Neurobehavioral assays were conducted 24 h before ICH and then repeated at 24, 48 and 72 h following ICH. Results: NOD mice showed increased hematoma volume and impairment in neurological function, as revealed by rotarod and grip strength analyses. Immunohistochemical staining showed reduced glial cell activation, as indicated by decreased GFAP and Iba1 staining. Furthermore, the expression of oxidative/nitrosative stress markers represented by 3-nitrotyrosine and inducible nitric oxide synthase was reduced in the diabetic group. Conclusions: Overall, our findings support the notion that hyperglycemia exacerbates ICH injury and worsens neurological function and that the mechanism of injury varies depending on the type of diabetes model used.

## 1. Introduction

Acute intracerebral hemorrhage (ICH) is considered the worst type of stroke, with a death rate of 30–40% and a low recovery rate following an attack [1,2]. Around 2 million ICH cases are reported worldwide annually, a figure that is expected to double by 2050 [3,4]. Ruptured blood vessels in the brain parenchyma and extravasation of the blood contribute to the pathogenesis of brain injury following ICH. Hematoma degradation products such as hemoglobin (Hb), heme, and iron trigger secondary brain injury via multiple mechanisms, including oxidative stress, excitotoxicity, and neuroinflammation [5]. Oxidative stress is characterized by the formation of reactive oxygen species such as superoxide and peroxynitrite, which cause cell death primarily by the oxidation of various cellular constituents. Thrombin, a clotting factor accumulated at high levels within the hemorrhagic area, activates endothelial cells, leading to blood–brain barrier (BBB) disruption and edema formation [6]. Additionally, it induces microglial cell activation and apoptosis of neurons and astrocytes [7,8]. Altogether, dead neurons and glia triggered by thrombin and hematoma degradation products release danger-associated molecular patterns (DAMPs) like adenosine triphosphate (ATP), neurotransmitters, nucleic acids, and high-mobility group box 1 proteins, which can also activate microglia, creating what is called self-perpetuating neurotoxicity [9,10].

Epidemiological studies have demonstrated that diabetes is a recognized independent and modifiable risk factor for ICH [11]. Regardless of the history of diabetes, hyperglycemia is often detected in 60% of patients on admission and within 72 h following ICH [12]. Importantly, persistent hyperglycemia following ICH is independently linked to deterioration in neurological functions and early death [13,14]. Mechanistically, animal model studies have reported that hyperglycemia aggravates neuronal death, BBB disruption, and edema formation [15]. In general, numerous in vitro and in vivo studies have established the upregulation of the oxidative and inflammatory markers in mice brains with diabetes [16]. Moreover, several preclinical translational studies have reported the deleterious effect of diabetes on ischemic stroke injury and outcomes; however, the cellular, subcellular, and molecular effects of diabetes on ICH pathology are less understood. Our previous study demonstrated that streptozotocin-induced diabetes in mice aggravated ICH injury and impaired neurological function by activating glial cells and inducing oxidative and inflammatory parameters [17]. Though preclinical, clinical, and epidemiological studies have demonstrated the unfavorable impact of hyperglycemia on ICH outcomes, therapeutic approaches aimed at lowering glucose levels provide inconclusive results [18]. Therefore, an in-depth understanding of the molecular mechanisms of hyperglycemia-induced neurodegeneration and cognitive deficits using multiple experimental models of diabetes is necessary to mitigate the comorbid effect on ICH.

In the previous study, we utilized streptozotocin-induced diabetes as a model for type-1 diabetes mellitus (T1DM) to explore the impact of diabetes on ICH. However, the present study investigated the role of diabetes using a non-obese diabetic (NOD) mouse model of TIDM using collagenase-induced ICH injury. The impact of diabetes on hemorrhagic outcomes such as hematoma volume, neurobehavioral outcomes, and inflammatory and oxidative stress parameters were investigated.

## 2. Materials and Methods

### 2.1. Animals and Study Design

**Figure 1:**
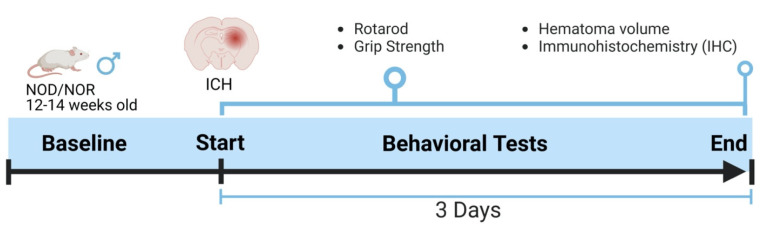
An outline showing the experimental design. The baseline readings for the neurobehavioral parameters were recorded day before ICH and then three days after that, just before euthanasia.

All animal protocols were approved by the Institutional Animal Care and Utilization Committee (IACUC) of the University of Toledo Health Science Campus under National Institute of Health guidelines. Mice were maintained in the Department of Laboratory Animal Resources (DLAR) at the University of Toledo with free food and water access, 21 °C temperature, and a 12 h light/dark cycle. NOD/LtJ mice were obtained from The Jackson Laboratory (Bar Harbor, ME, USA). NOD male mice were utilized in the present study and maintained in sterile conditions throughout the duration of the experiments. Mice were divided into two groups, diabetic and non-diabetic, and then subjected to ICH. Mice were sacrificed three days following the ICH, and brains were collected for further experiments. Figure 1 describes the experimental design used in this study.

### 2.2. The NOD Mouse Model of TIDM

NOD strain mice are albino immunodeficient mice that spontaneously develop diabetes through the leukocytic infiltration of pancreatic islets [19]. Age-matched males were determined to be diabetic (NOD) and non-diabetic (NOR) at 12 weeks. In sequential blood and urine measurements prior to ICH, hyperglycemia was confirmed by glucose levels > 300 mg/dL (188 ± 12 for NOR group, N = 14; 459 ± 18 for NOD group, N = 15) and re-evaluated prior to sacrifice. Animals were subjected to ICH after the confirmation of hyperglycemia, and neurobehavioral parameters were performed, as mentioned in Figure 1.

### 2.3. The ICH Model

Mice were subjected to ICH by stereotaxic intrastriatal injection of Collagenase VII-S (Sigma, St. Louis, MA, USA). In brief, mice were anesthetized by isoflurane and positioned on a stereotaxic instrument (Stoelting, Wood Dale, IL, USA). A 1 cm incision was performed on the head to expose the skull, and a 1 mm hole was manually drilled. The stereotaxic coordinates for collagenase injection were anteroposterior (AP) = 0 mm, mediolateral (ML) = 2.5 mm, and dorsoventral (DV) = 3 mm. Collagenase (0.5 µL, 0.075 U in normal saline) was injected slowly over 5 min, and the incision was sutured upon completion. The animals were allowed to survive for 72 h, during which period neurobehavioral parameters were measured daily. After 72 h, animals were deeply anesthetized by ketamine/xylazine and transcardially perfused with cold PBS, followed by 4% paraformaldehyde. Brains were dissected, cryoprotected with gradient sucrose solutions, and flash frozen at optimal cutting temperature (used as an embedding medium for cryotomy) for sectioning.

### 2.4. Neurobehavioral Parameters

NOD and NOR animals were trained on rotarod and grip strength tests for 3 days before the induction of ICH. Baseline values for each group were obtained 24 h before ICH, and subsequent measurements were normalized to their respective baselines. Measurements were taken in triplicate for each animal at each time point as follows: baseline, 24, 48, and 72 h.

Rotarod: Animals were positioned on the stationary rod of a rotarod instrument (Columbus Instruments, Columbus, OH, USA), which starts rotating at a speed of 4 rpm and accelerates at a rate of 1 rpm every 10 s until the animal falls from the rod. The time at which the animal fell was recorded manually as the latency to fall. Animals were subjected to three trials with five-minute intertrial intervals.

Grip Strength: The forelimbs of mice were positioned on the metal bar of the grip strength apparatus (Columbus Instruments), and mice were held by the tail by a researcher blind to the treatment groups and gently pulled until the animal released its grip. The peak force exerted by the mice was digitally recorded in Newtons (units of force) and noted.

### 2.5. Hematoma Volume Analysis

Frozen brains were sectioned for analysis of hematoma volume by cresyl violet (CV) and Luxol fast blue (LFB) staining, which stains the Nissl bodies and myelin of viable cells. Brains were sectioned into four 30 µm coronal sections spaced apart by 300 µm. Sections were stained with LFB and CV, dehydrated by solutions of increasing ethanol concentrations followed by xylenes, and then covered with a mounting medium and a coverslip. Pictures of stained sections were taken and analyzed by NIH Image J, version 1.53t, National Institute of Health, Bethesda, USA. The hematoma volume or area was defined as a lack of CV and LFB staining in the ipsilateral cortex. Next, 4–6 sections were taken for each animal and a researcher blind to the groups calculated the hematoma volume by summating the average area of two sequential sections multiplied by the distance between the sections (300 µM), as seen in the equation below:

(1) $$\sum \left(\frac{A_{n}+A_{n+1}}{2}\right)\times 300\;µm$$

### 2.6. Immunohistochemistry (IHC)

Frozen coronal sections (10 µm) were collected near the collagenase injection site and washed three times in PBS at room temperature (RT). Slides were deparaffinized and rehydrated with xylene/ethanol gradients, respectively. Slides were placed in a pressure cooker containing citrate buffer (pH 6.0) for 4 min for antigen retrieval. Tissues were permeabilized by 1% triton-X100 in PBS for 30 min, washed in PBS, and blocked in 1% BSA in PBS for 1 h at RT. Slides were incubated overnight at 4 °C in 1% BSA containing the following primary antibodies: mouse anti-3-NT (1:500, Sigma-Aldrich, St. Louis, MA, USA; Product No. SAB5200009), rabbit anti-iNOS (1:300, Cell Signaling Technology, Danvers, MA, USA; Product No. 95423), rabbit anti-GFAP (1:500, Abcam, Cambridge, UK; Product No. ab7260), and mouse anti-Iba1 (1:500, Abcam; Product No. ab283319). The slides were washed in PBS and then incubated for 1 h at RT in 1% BSA containing Alexa Fluor^®^594-conjugated goat anti-rabbit and DyLight™488-conjugated goat anti-mouse (1:500, Jackson ImmunoResearch, West Grove, PA, USA) secondary antibodies. After a final PBS wash, slides were mounted with SlowFade^®^ Diamond Antifade Mountant with DAPI (ThermoFisher, Waltham, MA, USA; Product No. S36967) and a coverslip was introduced on top. Images were captured using fluorescence microscopy.

### 2.7. Counting of Perihematomal Microglia and Astrocytes

Three coronal sections with 10 µm thickness were collected from each mouse and stained according to the above-mentioned IHC staining protocol. Iba1 and GFAP antibodies (Abcam) were used to visualize microglia and astrocytes. For the counting procedure, slides were magnified at 20× magnification, and three random points at the perihematomal area were chosen for cell counting. The perihematomal area was the surviving area surrounding the hematoma, which represented the core of bleeding, with no viable cells detected. For each animal, the cells from different sections were averaged and then analyzed with those of other animals (4–5 mice in each group) and plotted accordingly.

### 2.8. Statistical Analysis

Hematoma volume and IHC were analyzed using an unpaired *t*-test to compare the two groups. Neurobehavioral data were analyzed by two-way ANOVA with a Bonferroni post hoc test. Data were represented as mean ± SEM, with a value of *p* < 0.05 considered statistically significant.

## 3. Results

### 3.1. TIDM Exacerbates Neurological Outcomes at Late Time Points

**Figure 2:**
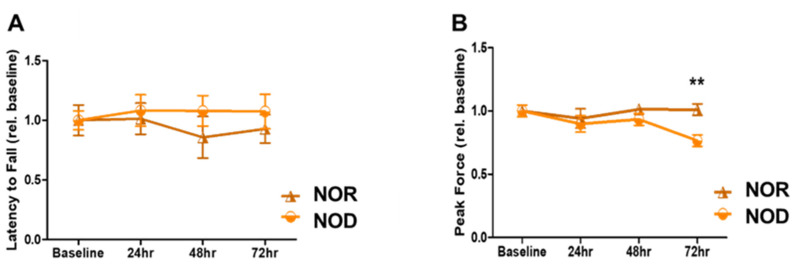
Neurobehavioral outcomes following ICH injury. (**A**) Rotarod was performed to assess motor coordination in mice by measuring the latency to fall in seconds at baseline and on days 1, 2, and 3 following the injury. (**B**) Grip strength was used to assess the forelimb grip strength at baseline and on days 1, 2, and 3 following the injury (N = 5 mice in each group). Data are expressed as mean ±  SEM (unpaired *t*-test), where *p*  <  0.01 represents significant **  differences between the represented groups.

Sensory and motor coordination were assessed using grip strength and rotarod assays. NOD and NOR mice were trained for three days before the ICH and then tested for another three days. There was no significant difference between the two groups in terms of rotarod performance over the three days (Figure 2A; *p* = 0.09 at 72 h after ICH). However, a primary difference between NOD and NOR mice was observed by the grip strength at 72 h after ICH, in which the NOR group performed significantly better than the NOD group (>20% difference, *p* < 0.01) (Figure 2B). These results indicate that TIDM negatively impacted the muscular strength following ICH.

### 3.2. NOD Mice Have Higher Hematoma Volume than NOR

**Figure 3:**
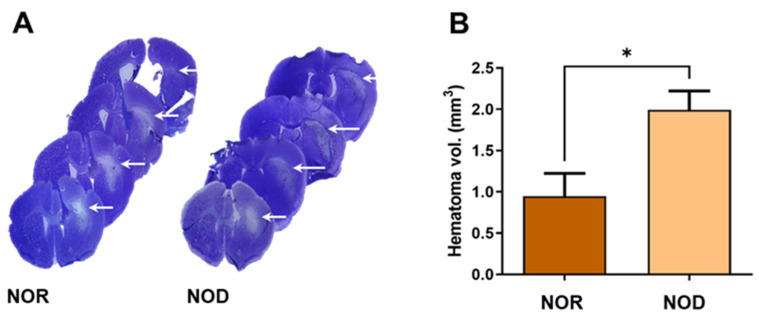
Assessment of the injury volume. (**A**) Brain sections were stained with cresyl violet (CV) and Luxol fast blue (LFB), which stains the Nissl bodies and myelin of viable cells. (**B**) Hemorrhagic volume analysis showed that NOD mice have significantly increased hematoma volumes compared to NOR mice (N = 4–5 mice in each group). Data are expressed as mean ±  SEM (unpaired *t*-test), where *p*  <  0.05 represents significance. *  Difference between the represented groups.  * *p*  <  0.05. White arrows show the hematoma areas.

Three days after ICH, frozen brain sections were stained with cresyl violet–Luxol fast blue staining to visualize the hematoma and perihematomal regions (Figure 3A). Analysis of the hemorrhagic injury volume revealed a significant increase in the hematoma volume with >100% larger hematoma in NOD compared to NOR mice (Figure 3B).

### 3.3. Glial Cell Activation Is Reduced in NOD Mice

**Figure 4:**
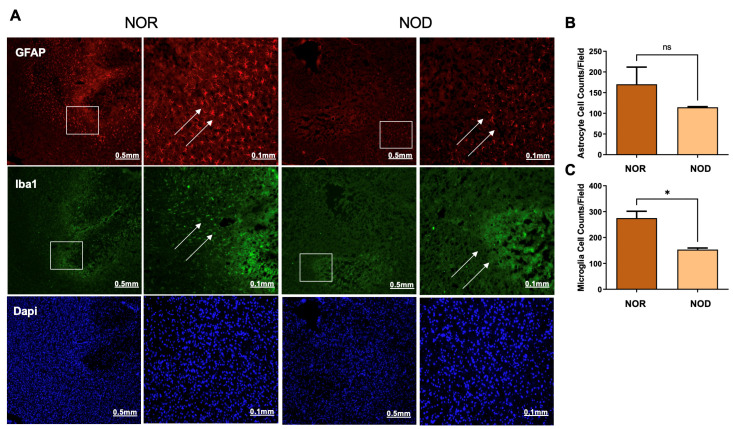
Glial cell activation at the perihematoma region (striatum) in mice 3 days following ICH injury. NOD mice show reduced microglial (**A**,**C**) and astrocytic (**A**,**B**) cell activation, as revealed by staining with rabbit anti-GFAP and mouse anti-Iba1, indicating a reduced inflammatory response for DM mice (N = 5 mice in each group). Data are expressed as mean ±  SEM (unpaired *t*-test), where *p*  <  0.05 represents significance. *  Difference between the represented groups. *  *p*  <  0.05; ns, non-significant. Boxes show the magnified areas (0.1 mm) and white arrows show the astrocytes and microglial cells in the perihematoma area.

Glial cells, including astrocytes and microglia, are activated from hours to days following ICH and are implicated in the secondary injury process. To explore the levels of glial cell activation in NOD and NOR groups, brain slices were stained with glial fibrillary acidic protein (GFAP) and ionized Ca^2+^-binding adapter molecule 1 (Iba1), markers of astrocyte and microglia activation, respectively. Interestingly, our results demonstrated that NOD mice exhibited a comparatively lower level of glial cell activation, represented by the fluorescence intensity, compared to NOR mice 3 days following ICH (Figure 4). Cell count analysis from different regions in the perihematomal areas showed a significant reduction in the microglial cells (Figure 4A,C). At the same time, there was a non-significant reduction trend in the astrocytes (Figure 4A,B).

### 3.4. Oxidative/Nitrosative Stress Is Reduced in NOD Mice

**Figure 5:**
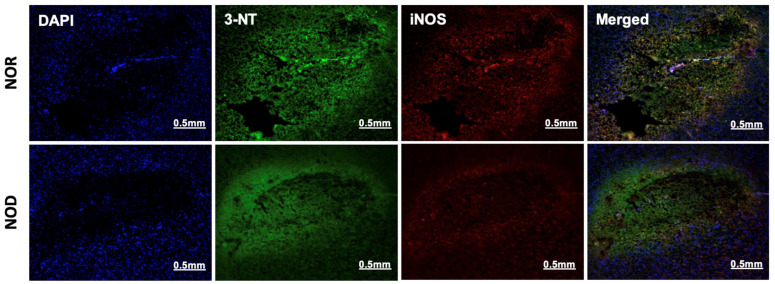
Oxidative/nitrosative stress response at the perihematoma region (striatum) in mice 3 days following ICH. NOD mice showed decreased iNOS and 3-NT expression, as revealed by rabbit anti-iNOS and mouse anti-3-NT specific antibodies, indicating a reduced immune response (N = 5 mice in each group).

The two phases of ICH, the primary and secondary injuries, are characterized by a free-radical burst and neuroinflammation, respectively. Immunohistochemistry was performed near the ICH region from each group to investigate the effects of the NOD models of T1DM on these pathologies. 3-nitrotyrosine (3-NT), a marker of peroxynitrite formation, and inducible nitric oxide synthase (iNOS) were used as an oxidative/nitrosative stress marker. iNOS is implicated in the generation of peroxynitrite under inflammatory conditions [20], and in the present study, its co-localization with 3-NT was observed in all mice subjected to ICH. Consistent with the glial cell activation results, immunohistochemical analysis of 3-NT and iNOS showed that NOD mice brains exhibited lower expression levels of both proteins and less co-localization than the NOR group (Figure 5).

## 4. Discussion

In the present study, a spontaneous, autoimmune-mediated NOD mouse model of T1DM was used to investigate the comorbid effect of diabetes after collagenase-induced ICH injury. Grip strength analysis performed at 72 h after ICH demonstrated that diabetic mice had a greater deficit in motor function than non-diabetic mice. However, no significant differences were detected in the rotarod performance. Furthermore, diabetic mice showed greater hematoma volume than non-diabetic. Contrary to the previously studied streptozotocin-induced model of diabetes, the current study demonstrated a reduction in the activation of glial cells, as well as in the oxidative/nitrosative stress markers, corroborating the differential effects of diabetic models (chemical-induced and immunological) on the pathology of ICH.

Current models of hyperglycemia and T1DM more closely replicate the underlying pathologies found in humans, effectively becoming more ‘humanized’ [21,22]. There are two commonly used but contrasting models of T1DM: acute induction by STZ administration and the spontaneous autoimmune-mediated NOD mouse model. Though each exhibits hyperglycemia and insulin deficiency due to β-cell destruction, the progression timeline to T1DM differs significantly between the models (5 days for STZ, 10 weeks for NOD) [23]. Due to the increase in susceptibility of T1DM patients to ischemic and hemorrhagic stroke, the application of these models of T1DM to ICH can help us to elucidate the causative factors unique to T1DM [11]. Identifying these factors and studying how they are modulated in T1DM may provide biomarkers for prevention, create new opportunities for intervention, and contribute to better therapies for non-diabetic patients.

Hyperglycemia is an independent predictor of poor outcomes in patients with acute ICH, increasing the risk of neurological damage and early mortality [24]. Although the precise pathophysiological mechanisms underpinning hyperglycemia-induced neurological deterioration in ICH remain unclear, it has been reported that hyperglycemia causes neuronal cell death, which manifests as neurobehavioral deficits in the behavioral assays [25]. In agreement with the previous reports, our findings demonstrated that the NOD group exhibited more impairment in neurological function than the NOR group following ICH, as manifested by a decline in the grip strength performance test. This can be explained by the fact that diabetes weakens grip strength by reducing skeletal muscles’ contractile force and increasing fatigability by affecting the total muscle fiber area, as observed in diabetic rats [26]. Therefore, in this study, there was a significant reduction in force strength in the NOD group but not in the NOR group. Similarly, the NOD group exhibited a larger hematoma volume relative to the control, indicating that ICH injury is more severe in the diabetic group. These findings corroborate the strong correlation between hematoma volume and performance in the neurobehavioral tests [27].

The influence of hyperglycemia on the activation of microglia and astrocytes, the prominent glial cells in the brain, is debatable. Some reports have demonstrated hyperglycemia-induced glial cell activation and proliferation with a profound increase in the secretion of proinflammatory cytokines, chemokines, and reactive oxygen and nitrogen species, exacerbating neuroinflammation and brain damage [28,29,30]. However, other reports demonstrated that hyperglycemia inhibited microglia and astrocyte activation [31,32,33]. It has been hypothesized that the concurrence of hyperglycemia and ischemia provokes an acidosis state, which damages astrocytes and inhibits ischemia-induced astrocyte activation [32,34]. Furthermore, another study has reported that hyperglycemia changes astrocyte metabolism and inhibits astrocyte proliferation [33]. In parallel with the later reports and contrary to our previous findings, the NOD group showed a reduction in the activation of microglia and astrocytes in the perihematomal area relative to the NOR group. These findings are well correlated with the reductions in the oxidative stress markers iNOS and 3-NT in the perihematomal area. To the best of our knowledge, our present and previous studies are the only ones that have investigated hyperglycemia’s impact on glial cell activation during the setting of ICH; however, enough data are available on other neurological diseases [35]. The variations in the activation status of glial cells by hyperglycemia during different disease settings could be attributed to differences in diabetes models, neurological disease models, and the duration and intensity of hyperglycemia.

An increasing amount of evidence has demonstrated that hyperglycemia increases the expression of inflammatory and oxidative stress markers such as proinflammatory cytokines, iNOS, and reactive oxygen species, which correlate with disease progression and decline in neurological function [36]. Oxidative stress and inflammation are inextricably linked and have been identified as critical contributors to ICH-induced injury by promoting cytotoxicity and brain edema [37,38]. In the rat model of focal ischemia, hyperglycemia augmented superoxide production in brain parenchyma and vasculature, contributing to BBB disruption and increasing the risk of tPA-induced hemorrhage [39]. In our previous study of STZ-induced T1DM in mice, hyperglycemia increased iNOS and 3-NT expression in the perihematomal area following ICH [17]. Contrary to all the previously mentioned reports, NOD mice exhibited lower expression of iNOS and 3-NT than NOR littermates, as illustrated by immunohistochemical staining analysis. During inflammatory reactions in the brain, iNOS is highly expressed by microglia, astrocytes, and vascular cells [40,41]. However, our findings correlated to reductions in activated astrocytes and microglia in the perihematomal regions of NOD animals compared to the NOR group. The reason for this decrease in inflammatory response was not clear, and it warrants further investigations. NOD mice have compromised innate immune systems and exhibit reduced NK cells and macrophage activity [42]. Compared to control macrophages, NOD mouse macrophages showed a significant impairment in the phagocytosis of apoptotic cells [43]. Thus, it is unclear whether this immunological deficit also impacts microglia. Moreover, NOD mice develop spontaneous autoimmunity against pancreatic β cells, resulting in insulitis 3–4 weeks after birth. However, hyperglycemia is identified at the age of 12–14 weeks, which is different from the pattern of hyperglycemia induced by streptozotocin [44]. It is currently unclear whether the reduced glial cell activation and proliferation were driven by defective innate immunity or variations in hyperglycemia development patterns between the two models.

The extent of brain damage following ICH injury is an outcome of the possible contribution of the following well-known pathophysiologic mechanisms: oxidative stress, inflammation, and excitotoxicity. However, the contribution of each one of these mechanisms to the overall damage is yet to be determined. Importantly, the exact molecular mechanisms underpinning hyperglycemia’s detrimental effects in ICH are still unclear. One of the proposed mechanisms underlying hyperglycemia-associated brain injury might be through inducing neuronal apoptosis [25]. Another suggested mechanism is the downregulation of aquaporin-4, which aggregates ICH injury by inducing BBB disruption and brain edema [45]. Further investigations are required to prove these mechanisms.

According to the previously mentioned results, this work has several limitations, and future studies are required to fill the gaps in the presented research. It is still unclear how hyperglycemia exacerbates ICH injury, and this warrants further investigations to decipher the molecular mechanisms linking hyperglycemia to glial cell activation, oxidative stress, and neuroinflammation. The other limitation of this study was that it is unknown whether hyperglycemia or a defective immune system plays the primary role in suppressing glial cell activation and oxidative stress at the perihematomal area. Therefore, investigating different inflammatory and oxidative stress markers during the acute phase of ICH is required to gain more insights into the mechanism of hyperglycemia-induced brain injury. Additionally, investigating the long-term effects of hyperglycemia on brain injury and functional recovery supported by different and sufficient neurobehavioral assays is necessary to find more detail about the comorbid impact of diabetes on ICH pathophysiology. It is still unknown whether the degree of damage following ICH is higher in the STZ or NOD models, suggesting that a comparative and parallel study to is required to find more details about the differential effects of each model on the pathophysiology of ICH.

## 5. Conclusions

The present study provides clear insights into the effect of diabetes on collagenase-induced ICH in NOD mice. Hyperglycemia aggravates brain injury and worsens the neurobehavioral deficits following ICH. However, it is still unclear whether hyperglycemia or a defective immune system contributes mostly to dampened glial cell activation and oxidative stress following ICH. Furthermore, the molecular mechanism underlying hyperglycemia-induced brain injury following ICH in NOD mice remains unclear and warrants further investigation. Therefore, additional studies are warranted to gain a deep understanding of the comorbid effect of diabetes on ICH injury.

## Data Availability

Data is contained within the article.
